# P2Y12 Receptor Inhibitor for Antiaggregant Therapies: From Molecular Pathway to Clinical Application

**DOI:** 10.3390/ijms25147575

**Published:** 2024-07-10

**Authors:** Francesco Nappi

**Affiliations:** Department of Cardiac Surgery, Centre Cardiologique du Nord, 93200 Saint-Denis, France; francesconappi2@gmail.com or f.nappi@ccn.fr; Tel.: +33-149334104; Fax: +33-149334119

**Keywords:** platelets, antiplatelet drugs, P2Y12 inhibitor, thrombosis, bleeding

## Abstract

Platelets play a significant role in hemostasis, forming plugs at sites of vascular injury to limit blood loss. However, if platelet activation is not controlled, it can lead to thrombotic events, such as myocardial infarction and stroke. To prevent this, antiplatelet agents are used in clinical settings to limit platelet activation in patients at risk of arterial thrombotic events. However, their use can be associated with a significant risk of bleeding. An enhanced comprehension of platelet signaling mechanisms should facilitate the identification of safer targets for antiplatelet therapy. Over the past decade, our comprehension of the breadth and intricacy of signaling pathways that orchestrate platelet activation has expanded exponentially. Several recent studies have provided further insight into the regulation of platelet signaling events and identified novel targets against which to develop novel antiplatelet agents. Antiplatelet drugs are essential in managing atherothrombotic vascular disease. The current antiplatelet therapy in clinical practice is limited in terms of safety and efficacy. Novel compounds have been developed in response to patient variability and resistance to aspirin and/or clopidogrel. Recent studies based on randomized controlled trials and systematic reviews have definitively demonstrated the role of antiplatelet therapy in reducing the risk of cardiovascular events. Antiplatelet therapy is the recommended course of action for patients with established atherosclerosis. These studies compared monotherapy with a P2Y12 inhibitor versus aspirin for secondary prevention. However, in patients undergoing percutaneous coronary intervention, it is still unclear whether the efficacy of P2Y12 inhibitor monotherapy after a short course of dual antiplatelet therapy depends on the type of P2Y12 inhibitor. This paper focuses on the advanced-stage evaluation of several promising antiplatelet drugs.

## 1. Introduction

Antiplatelet therapy and platelet signaling pathways play a crucial role in the treatment of coronary artery disease (CAD) and percutaneous coronary intervention (PCI). The introduction of new inhibitors has facilitated enhanced treatment options. It is of paramount importance to adhere rigorously to established metrics and units when measuring the effectiveness of treatment.

Over the past decade, there has been a notable expansion in our comprehension of the signaling pathways that activate platelets [1,2,3,4]. The aforementioned proteins and agonists initiate signaling transduction through interaction with the corresponding receptors. The signaling pathways that activate platelets converge into common events that stimulate shape change and granule secretion. These events are specific to each receptor. The process in question activates the ligand-binding function of integrin α(IIb)β [3]. Ligand binding then mediates platelet adhesion and aggregation. This occurs through the triggering of outside-in signaling with confidence. Platelet activation results in a series of events, including spreading, granule secretion, adhesion, aggregation stabilization, and clot retraction. Agonist-induced activation of platelets, in conjunction with signals initiated by integrins on the platelet surface, regulate platelet responses in a coordinated manner. 

This process involves the rapid formation of positive feedback loops that serve to amplify activation signals. This allows for enhanced platelet recruitment and stabilization of the thrombus [1]. 

It is evident that antiplatelet therapies, such as aspirin, clopidogrel, and prasugrel, play an essential role in the treatment of patients at high risk of arterial thrombosis [5,6,7,8,9]. It can be demonstrated that antiplatelet agents have a significant beneficial effect in reducing morbidity and mortality due to vascular events by limiting platelet activation in patients who are at risk of developing arterial thrombosis [10]. While these medications are associated with a significant risk of bleeding, these medications remain an important tool in preventing arterial thrombotic events. To identify safer targets for antiplatelet therapy, a better understanding of platelet signaling mechanisms is necessary [11]. Although there are restrictions on their usage, some inherent limitations may restrict the applicability of these techniques in specific contexts.

In accordance with current clinical guidelines, dual antiplatelet therapy (DAPT) with aspirin and a P2Y12 inhibitor represents the recommended course of action following PCI, with the goal of reducing the risk of subsequent cardiovascular ischemic events. Nevertheless, prolonged DAPT use is associated with an increased risk of bleeding. Studies employing an abbreviated duration of DAPT have reported a reduction in bleeding, but an increase in ischemic risk, particularly in patients presenting with acute coronary syndrome (ACS) or complex PCI, in comparison to the standard duration of DAPT. More recently, the cessation of aspirin and the continuation of the P2Y12 inhibitor following a brief course of DAPT have been subjected to further investigation.

The evidence indicates that the duration of DAPT followed by aspirin monotherapy is associated with a lower risk of bleeding, but a higher risk of ischemic events, particularly in patients with acute coronary ACS or complex PCI, compared with the standard duration of DAPT [12,13,14].

The recent investigations have focused on the cessation of aspirin and the continuation of the P2Y12 inhibitor following a brief course of DAPT [15,16,17,18,19,20]. A patient-level meta-analysis comprising 23,308 subjects undergoing coronary revascularization indicated that P2Y12 inhibitor monotherapy, following a 1- to 3-month course of DAPT, exhibits equivalent safety for patients while conferring a benefit in terms of reduced risk of major bleeding in comparison to conventional DAPT [21].

It is notable that the antiplatelet activity of clopidogrel exhibits considerable interindividual variation. The small sample size of patients receiving clopidogrel monotherapy limits the ability to draw definitive conclusions on how efficacy may vary based on the specific P2Y12 inhibitor. The available evidence clearly demonstrates that clopidogrel is associated with significant variability in platelet response between patients. It is estimated that up to 30% of patients undergoing treatment display high residual platelet reactivity, which is associated with an increased risk of subsequent cardiovascular events [22]. Conversely, prasugrel represents a more efficacious therapeutic alternative for patients exhibiting diverse CYP2C19 phenotypes [23]. Previous research has demonstrated that the CYP2C19 phenotype has no impact on the response to prasugrel, but plays a significant role in the variability of response to clopidogrel [23,24,25]. Furthermore, the results demonstrated that prasugrel exhibited higher active metabolite exposure and a more consistent pharmacodynamic response across all three predicted phenotype groups in comparison to clopidogrel [23,24,25]. A 2022 randomized clinical trial (RCT), involving 4169 patients presenting with ACS who underwent implantation of current-generation drug-eluting stents (DES), demonstrated that clopidogrel monotherapy following one to two months of DAPT did not exhibit inferiority to conventional DAPT in terms of net clinical benefit. Furthermore, clopidogrel was associated with a significant increase in the incidence of myocardial infarction (MI) [26].

**Figure 1:**
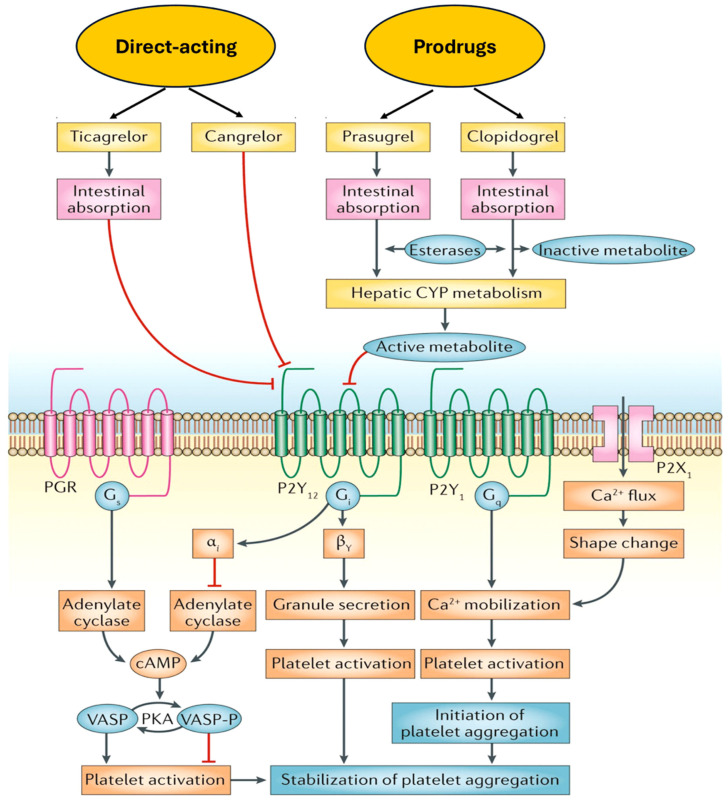
Mechanism of action and metabolism of P2Y12 receptor inhibitors. P2Y12 receptor inhibitor therapies act directly or through prodrugs that generate active metabolites. Both platelet activation and platelet aggregation stabilization are effectively blocked by these therapies. Abbreviations: AMP, adenosine monophosphate; CYP; Cytochrome P450; P2Y; purinergic G protein-coupled receptors; PGR, progesterone receptor; PKA; protein kinase A; VASP; vasodilator-stimulated phosphoprotein [1,2,3,4,5,6,7,8,9,10,11,12,13,14,15,16,17,18,19,20,21,22,23,24,25,26,27].

A recent patient-level meta-analysis from Valgimigli et al. [5] definitively demonstrated that ticagrelor monotherapy was non-inferior to DAPT for all-cause death, MI, or stroke, and superior for major bleeding and net adverse clinical events (NACEs). Clopidogrel monotherapy also reduced bleeding. Nevertheless, the analysis revealed no evidence of non-inferiority with regard to all-cause death, MI, or stroke compared to DAPT. This discrepancy can largely be attributed to a specific risk observed in a single clinical trial, which exclusively included East Asian participants. This was driven by a higher incidence of non-cardiovascular deaths [5]. Moreover, a fundamental assessment of the pharmacodynamic data shows that in contemporary real-world ST elevation myocardial infarction (STEMI) patients pretreated within 1 h with ticagrelor undergoing primary PCI, adding cangrelor resulted in fast and potent platelet inhibition. This suggests that cangrelor may bridge the gap until ticagrelor reaches its effect [27] (Figure 1).

## 2. Search Strategy and Selection Criteria

PubMed, MEDLINE, and Embase were thoroughly searched using the terms ‘antiplatelet drugs’, ‘antiplatelet therapies’, ‘antiplatelet medications’, ‘clopidogrel’, ‘prasugrel’, ‘ticagrelor’, ‘percutaneous coronary intervention’, ‘myocardial infarction’, combined with ‘prevention’, ‘diagnosis’, and ‘treatment’. The selected articles were primarily RCTs, meta-analyses, propensity studies, and research studies published within the last 15 years, until March 2024. Older publications, which are commonly referenced and highly regarded, from RCTs, observational studies, and reviews, were not excluded. The articles were categorized based on risk factors, prevention, classification, clinical diagnosis, immediate management, treatment strategies, and long-term follow-up.

Clinical status that requires P2Y12 receptor inhibitor therapies presents a unique challenge due to the need for both urgent and delayed treatment. Fortunately, a large body of randomized studies has determined the optimal treatment, enabling confident decision-making. Table 1 provides a clear illustration of the antiplatelet drugs that have been subjected to the most comprehensive analysis in recent meta-analyses and RCTs, and those that are most closely aligned with clinical applications [5,6,7,8,9,13,14,15,16,17,18,19,20,21,22,26,27,28,29,30,31,32,33,34,35,36,37,38,39,40,41].

## 3. Purinergic Receptors’ Mechanism of Action: Thienopyridine vs. Nonthienopyridine P2Y12 Inhibitors

One of the fundamental tenets of contemporary antiplatelet therapy is the inhibition of ADP-mediated platelet activation and aggregation via the P2Y12 receptor. A significant obstacle to the efficacy of thienopyridine-based P2Y12 inhibitors, including clopidogrel and prasugrel, lies in the occurrence of heightened on-treatment platelet reactivity, which can be defined as a greater than anticipated platelet response to agonists [37,38,39,40,41]. A recent report has highlighted the significant role of platelet turnover in the context of high on-treatment platelet reactivity. In vitro and ex vivo studies have indicated that the limited half-life of clopidogrel and prasugrel, when combined with inadequate inhibition of aggregation of newly generated platelet reticular conglomerates upon ADP stimulation, may result in suboptimal antiplatelet effects [37]. In contrast, nonthienopyridine P2Y12 inhibitors, such as ticagrelor, demonstrated sustained platelet inhibition even in the absence of pretreated platelet populations [37].

### Effective Signaling Pathways and Platelet Inhibitory Purinergic Receptors

The adhesion, activation, and aggregation of platelets on the subendothelial extracellular matrix (ECM) are essential for hemostasis. The glycoprotein Ib-IX complex, also known as GPIb-V-IX, binds to immobilize von Willebrand factor (VWF), thereby initiating the adhesion of flowing platelets to the ECM. Furthermore, glycoprotein VI (GPVI) interacts with its ligand and effectively mediates platelet activation. The local production of thrombin and platelet-derived secondary mediators, such as ADP and TxA [2], exerts a profound influence on platelet adhesion and aggregation. This process is initiated by a change in the affinity state of the integrins, specifically the β1 and β3 integrins, for their ligands. This change occurs as a result of a signaling process referred to as ‘inside-out signaling’. The process of fibrin formation and the signaling events that take place between adjacent platelets are stabilized by the formation of platelet aggregates. This process involves the participation of multiple platelet receptors [2].

The recently discovered C-type lectin-like receptor 2 (CLEC-2) is a receptor that exhibits unique characteristics. Myocardial infarction is primarily the consequence of occlusive thrombus formation. Conversely, ischemic stroke presents a more complex situation. Nevertheless, even in the event of prompt recanalization of previously occluded major cerebral arteries, the progression of infarction frequently continues. The aforementioned process is referred to as reperfusion injury. In this setting, early platelet adhesion and activation events initiate a thrombo-inflammatory cascade. The formation of platelet aggregates and thrombi is not a prerequisite for the development of reperfusion injury [2].

The function of platelets is contingent upon the equilibrium of activating and inhibitory signaling pathways, which are responsible for the processes of hemostasis and thrombosis. This equilibrium ensures the efficacious and regulated functioning of hemostasis and thrombosis. It is crucial to recognize that maintaining this delicate balance is of paramount importance for the optimal functioning of hemostasis and thrombosis. Nevertheless, inhibitory stimuli emanating from the healthy vasculature are capable of effectively suppressing platelet activation in the absence of platelet receptor agonists. In the context of an injury, activating signals are initiated, leading to platelet activation and thrombus formation. Endogenous negative signaling regulators then serve to effectively dampen activating signals in order to control thrombus growth.

The comprehension of the intricate interrelationship between activating and inhibitory signaling networks represents a burgeoning challenge in the field of platelet biology, necessitating the implementation of a meticulous approach to the utilization of experimental data. The signaling pathways of platelets will be described in terms of the key signaling molecules that are common to the pathways activated by platelet agonists. These molecules can be described as regulatory nodes for both positive and negative regulators [3].

The activation signaling pathways of platelets are intricate and sophisticated. The discovery of novel receptor signaling pathways and regulatory networks has led to the questioning of some long-standing concepts of platelet signaling. Further research in this field is essential to achieve a comprehensive understanding of the complexities of platelet activation [4].

Currently, the thienopyridine-based P2Y12 inhibitors, including clopidogrel and prasugrel, are utilized for the purpose of targeting ADP-mediated platelet activation and aggregation via the P2Y12 receptor. Nevertheless, one of the principal obstacles confronted by these inhibitors pertains to the phenomenon of elevated on-treatment platelet reactivity. This is defined as a platelet response to an agonist that exceeds expectations [36].

The findings of Armstrong et al. [37] provide compelling evidence that platelet turnover plays a significant role in high on-treatment platelet reactivity. The relatively short half-life of clopidogrel and prasugrel resulted in an insufficient level of inhibition of platelet aggregation in newly formed, reticulated platelets upon ADP stimulation, as evidenced in both in vitro and ex vivo study models. Further research is necessary to determine whether there is a clinical benefit of nonthienopyridine-based P2Y12 inhibitors in the context of high on-treatment platelet reactivity. Despite the good level of platelet inhibition maintained by ticagrelor, a nonthienopyridine-based P2Y12 inhibitor, even when untreated platelets are introduced, it is unclear whether there is any clinical benefit of nonthienopyridine-based P2Y12 inhibitors in the context of high on-treatment platelet reactivity [37].

GLS-409 represents an emerging diadenosine tetraphosphate derivative that exhibits potential as a novel antiplatelet agent. Its dual targeting of the P2Y12 receptor and the second human platelet ADP receptor, P2Y1, suggests that it may offer a unique profile in the treatment of cardiovascular diseases. The in vivo antithrombotic effects of GLS-409 were evaluated in a study conducted by Gremmel and colleagues [38]. This study represents the first investigation of this particular antiplatelet agent.

The effects of GLS-409 were evaluated in anesthetized rats using a variety of methodologies. These included assessment of its effects on agonist-stimulated platelet aggregation, as well as its antithrombotic activity, which encompassed its impact on bleeding time in a canine model of platelet-mediated coronary artery thrombosis. The findings indicate that GLS-409 is an effective agent for reducing platelet aggregation and preventing thrombosis. Furthermore, the study aimed to compare the inhibitory effect of GLS-409 on agonist-stimulated platelet aggregation to that of selective P2Y1 and P2Y12 inhibition in vitro, using samples from healthy human subjects before and 2 h after aspirin intake [38].

GLS-409 demonstrated efficacy in inhibiting platelet aggregation in rats stimulated by adenosine diphosphate and collagen. Moreover, GLS-409 was found to successfully attenuate cyclic flow variation, a platelet-mediated thrombosis, in our canine model of unstable angina. The improvement in coronary patency was accompanied by a slight, non-statistically significant increase of 30% in the bleeding time, which may be attributable to a number of factors. It is crucial to highlight that GLS-409 achieved these outcomes without any adverse effects on rat and canine hemodynamics. In vitro studies have indicated that GLS-409 has similar effects on agonist-stimulated platelet aggregation to those of cangrelor and the combination of cangrelor with the selective P2Y1 inhibitor MRS 2179. These effects were observed in human platelet-rich plasma and whole blood, with the administration of GLS-409 prior to and 2 h after aspirin. Inhibition of the P2Y1 and P2Y12 adenosine diphosphate receptors by GLS-409 results in the attenuation of platelet-mediated thrombosis and the blocking of agonist-stimulated platelet aggregation, irrespective of the concomitant administration of aspirin therapy [38].

Platelets express both P2Y12 and P2Y1 receptors, with the latter being responsible for regulating the initial mobilization of calcium ions and the shape change of the platelets. It is crucial to address both receptors in order to achieve comprehensive regulation of platelet activation. The Ca^2+^ P2Y1 receptor is responsible for the initial mobilization of calcium ions and for regulating their concentration with great precision. Nevertheless, the current therapeutic approaches solely address P2Y12, leaving P2Y1-mediated platelet activation unaddressed. Yanachkov et al. demonstrated that GLS-409 effectively inhibits both P2Y1 and P2Y12 [39]. The derivatives were subjected to testing to ascertain their effects on platelet aggregation and the function of the platelet P2Y1, P2Y12, and P2 × 1 receptors. The previously established structure–activity relationships were employed to synthesize Ap4A analogs via in vitro methods. These analogs were found to exhibit inhibitory effects on human platelet aggregation, specifically by concurrently targeting the P2Y1 and P2Y12 platelet receptors. In comparison to Ap4A, the analogs display superior characteristics in several respects. Furthermore, they do not activate platelet P2X1 receptors, and their onset and offset of action are faster than those of Ap4A. Moreover, they exhibit greater stability in plasma than Ap4A, which renders them a highly promising new class of antiplatelet agents [39].

The human platelet population expresses three purinergic receptors: P2Y12, P2Y1, and P2X1. Collectively, these receptors orchestrate pivotal steps in the activation and aggregation of platelets. The prevailing therapeutic strategy for patients presenting acute coronary syndrome or undergoing percutaneous coronary intervention involves the selective blockade of the platelet P2Y12 adenosine diphosphate (ADP) receptor and thromboxane production inhibition via aspirin [40].

Despite the advent of percutaneous coronary intervention (PCI) techniques that aim to reduce excessive platelet function in high-risk settings, complications such as stent thrombosis and major bleeding can still occur. These complications remain a significant cause of morbidity and mortality. It is crucial to acknowledge that patients who have experienced an acute coronary syndrome may exhibit delayed or inconsistent metabolism of orally administered antiplatelet agents. Acute coronary syndrome is associated with an elevation in the proportion of young and immature platelets, which results in heightened platelet hyperactivity and an increased risk of thrombosis [41].

The continued need for fast-acting, potent, and safe antiplatelet agents in modern clinical practice is evident. While traditional platelet targets may be adequately inhibited by traditional antiplatelet agents, there is a possibility that other surface receptors and intracellular signaling pathways may still be activated.

GLS-409 demonstrated efficacy in inhibiting platelet aggregation and preventing recurrent coronary thrombosis in a canine model [38]. Additionally, GLS-409 was found to have a negligible effect on bleeding time. These results demonstrate the efficacy and safety of GLS-409 as a potential treatment for coronary thrombosis. It is conceivable that dual P2Y1/P2Y12 antagonists may eventually replace classical unimodal P2Y12 inhibitors in clinical use [40].

Gremmel et al.’s [39] investigation of a novel approach to ADP antagonism represented a pivotal advancement in the field of antiplatelet therapies. This approach will overcome the obstacles and provide complete and consistent platelet inhibition. In order to test the effectiveness of the modified nucleotide analogue GLS-409 in inhibiting the platelet aggregation process, the researchers conducted a study. The results demonstrated that the compound utilizes its inherent biological antiplatelet properties to simultaneously block the P2Y12 and P2Y1 receptors, offering a promising avenue for future research. The investigators conducted a series of meticulously designed experiments, each progressively smaller in scale, which significantly enhanced our understanding of the pharmacokinetic and pharmacodynamic properties of this prototypical agent. The initial study demonstrated that the administration of GLS-409 in vivo resulted in a robust inhibition of platelet aggregation in rats. In a canine model of recurrent coronary thrombosis, GLS-409 demonstrated the ability to improve selected coronary blood flow metrics, although this was accompanied by a prolongation of the bleeding time. In vitro studies conducted on blood samples obtained from healthy human volunteers demonstrated that GLS-409 demonstrated platelet aggregability comparable to that of cangrelor alone and to a combination of cangrelor and a selective P2Y1 inhibitor, including after the administration of aspirin at a high dose. These findings indicate that GLS-409 may possess antiplatelet properties, yet further investigation is warranted to ascertain its safety and efficacy [39]. The results of the study indicate that the novel compound under investigation is safe for the cardiovascular system. The stability of rat and canine hemodynamics provides evidence of this conclusion.

Nevertheless, it is necessary to conduct further clinical studies in order to fully evaluate the effects of adjunctive P2Y1 receptor inhibition. The preliminary findings indicate that further research in this area is necessary [11]. It can be expected that diadenosine tetraphosphate analogs will prove to be clinically effective in the reduction in ischemic risk while maintaining an acceptable safety profile. It is of the utmost importance to monitor patients for potential off-target effects, as P2Y1 receptors have been found to be expressed in a multitude of tissues beyond the platelet surface [42,43].

This novel class of platelet antagonists is administered intravenously, exhibiting rapid onset and reversibility. The pharmacological profile of this agent is highly appealing to patients at high risk for coronary thrombosis and major bleeding. GLS-409 and other diadenosine tetraphosphate derivatives are anticipated to undergo further preclinical and clinical development with the objective of confirming their efficacy, safety, practical applicability, and incremental value. The pharmacological properties observed in vivo are analogous to those observed with cangrelor, a potent intravenous P2Y12 ADP antagonist that has recently received approval for use during percutaneous coronary intervention in the United States and Europe [44].

Inhibiting P2Y12 represents a promising therapeutic strategy for sepsis. In a model of sepsis-induced inflammation, treatment with clopidogrel demonstrated the capacity to reduce the aggregation of platelets, the formation of platelet–leukocyte aggregates, and the extent of lung injury in both wild-type and P2Y12-deficient mice. These observations indicate that clopidogrel might be a viable option for the treatment of sepsis-induced inflammation [45].

In a study conducted by Liverani et al. [46], the role of the P2Y12 receptor in neutrophil migration and lung inflammation was investigated in a mouse model of intra-abdominal sepsis and acute lung injury. In order to ensure the reliability of the results, the researchers utilized P2Y12-null mice and mice pretreated with the P2Y12 antagonist clopidogrel. The results indicate a notable reduction in circulating white blood cells, platelet activation, and platelet–leukocyte interactions in mice treated with the P2Y12 antagonist clopidogrel compared to untreated mice. The administration of clopidogrel to mice with sepsis resulted in a reduction in lung injury and platelet sequestration when compared to untreated septic littermates. In septic P2Y12-null mice, similar results were obtained, with a reduction in both platelet activation and platelet–leukocyte aggregates compared to the wild-type mice. Furthermore, P2Y12-null mice exhibited a reduced susceptibility to lung injury in comparison to wild-type mice. Moreover, a pretreatment was performed on P2Y12-null mice to evaluate the effects of clopidogrel in mice that lacked the P2Y12 receptor, thereby eliminating potential confounding effects of the P2Y12 receptor on the outcomes. The findings of the study are unequivocal in demonstrating that clopidogrel exerts pleiotropic effects on neutrophils in septic mice lacking the P2Y12 receptor, as evidenced by a significant reduction in the number of circulating neutrophils. The absence of this effect in P2Y1-null mice provides compelling evidence that the P2Y12 receptor is the sole mediator of these effects. It has been demonstrated that activated platelets and the P2Y12 receptor play a crucial role in sepsis-induced lung injury. This is evidenced by the fact that mice deficient in P2Y12 are resistant to this condition [46].

**Figure 2:**
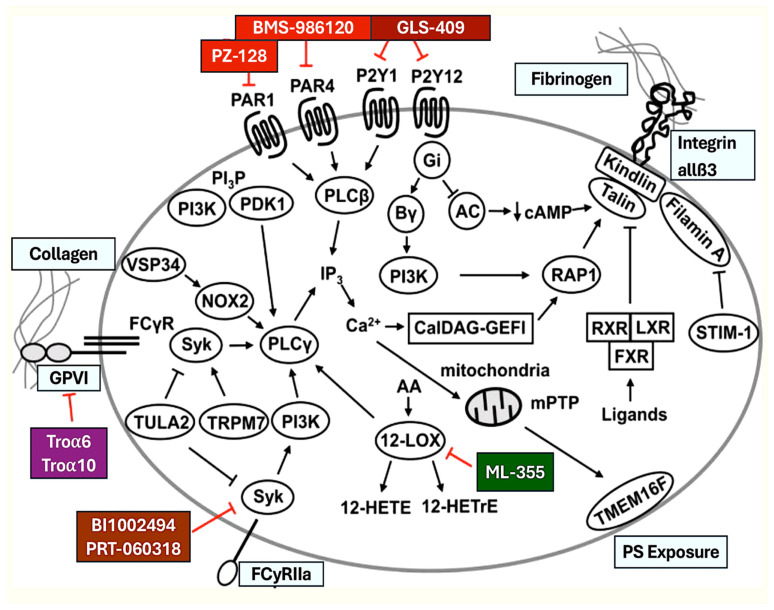
An in-depth overview of the intracellular platelet signaling pathways that are essential for platelet activation is provided. Additionally, the latest antiplatelet activators that are currently undergoing preclinical and clinical development are highlighted. The differently colored boxes indicate the new drugs and their respective targets. The uniformly colored boxes indicate the interactions between the different molecules and platelet receptors. Abbreviations, 12-HETE, 12(S)-hydroxy-5,8,10,14-eicosatetraenoic acid; 12-HETrE, 12S-hydroxy-8Z,10E,14Z-eicosatrienoic acid; 12-LOX, 12-lipoxygenase; AC, adenylyl cyclase; FCγR, Fcγ receptor; FXR, farnesoid X receptor; GPVI, glycoprotein VI; IP3, inositol 1,4,5-triphosphate; LXR, liver X receptor; mPTP, mitochondrial membrane transition pore; NOX2, nicotinamide adenine dinucleotide phosphate (NADPH Oxidase 2; PAR, protease-activated receptors; PDK1, phosphoinositide-dependent protein kinase 1; PI3K, phosphoinositide 3-kinase; PI3P, phosphatidylinositol 3-phosphate; PLC, phospho-lipase C; RAP1, Ras-related protein 1; RXR, retinoid X receptor; STIM, -1, stromal interaction molecule 1; Syk, spleen tyrosine kinase; TMEM16F, transmembrane protein 16F; Tro, trowaglerix; TRPM7, transient receptor potential melastatin-like 7; TULA2, T-cell ubiquitin ligand-2; and VPS34, vacuolar protein sorting 34 [2,3,4,37,38,39,40,41,42,43,44,45,46,47].

The response of platelets to septic challenge is primarily driven by the activation of P2Y12, as evidenced by the lack of protection observed in P2Y1-deficient mice [46]. These results are consistent with those of a previous study which demonstrated a reduction in neutrophil recruitment and lung injury in mice subjected to abdominal sepsis and treated with ticagrelor. Platelets contribute to sepsis-induced lung injury by enhancing neutrophil recruitment. In another experiment, wild-type C57BL/6 mice underwent cecal ligation and puncture (CLP), and were administered ticagrelor (100 mg/kg) or vehicle prior to CLP induction [47]. The findings of the flow cytometry analysis revealed a reduction in platelet–neutrophil aggregation, neutrophil activation, and CD40L expression on platelets in the ticagrelor-treated group. This indicated a pronounced protective effect of ticagrelor against CLP-induced lung damage. The quantification of edema formation, the bronchoalveolar neutrophil count, and the degree of lung injury demonstrated that the administration of ticagrelor significantly reduced the pulmonary infiltration of neutrophils induced by CLP at 24 h by 50% compared to a control group. Ticagrelor not only abrogated the edema of the lungs resulting from CLP, but also reduced the lung damage score by 41%. The administration of ticagrelor completely prevented the formation of platelet–neutrophil aggregates and significantly decreased thrombocytopenia in animals with CLP. Furthermore, ticagrelor was observed to reduce the shedding of CD40L from platelets in septic mice. Ticagrelor has been demonstrated to reduce pulmonary neutrophil recruitment and lung damage induced by CLP, suggesting a potential role for platelet antagonists, such as ticagrelor, in the management of patients with abdominal sepsis [47] Figure 2.

## 4. Current Therapies on P2Y12 Receptor Inhibitor

Platelets play a crucial role in forming hemostatic clotting cushions at sites of vascular damage to reduce blood loss. Antiplatelet agents are used clinically to inhibit platelet activation in patients at risk for arterial thrombotic events. The six most recent meta-analyses, which examined both trial-level and patient-level meta-analyses and were based on randomized clinical trials, highlighted the following major complications associated with the use of antiplatelet agents [5,6,48,49,50].

### 4.1. Assessing Meta-Analyses Investigating DAPT vs. SAPT for Balancing High Risk of Bleeding and Thrombosis

Valgimigli and colleagues conducted a patient level meta-analysis that clearly demonstrated the results of PCI studies [5] (Table 2). The analysis consisted of six randomized, controlled trials involving 25,960 patients who underwent PCI [15,16,17,18,19,26]. A one-step mixed-effects model was used to analyze the pooled dataset, synthesized from patient-level data from each trial. The per-protocol analysis included 24,394 patients: 12,403 were given DAPT, 8292 received ticagrelor monotherapy, 3654 were given clopidogrel monotherapy, and 45 patients were given prasugrel monotherapy. Ticagrelor monotherapy trials were conducted across Asia, Europe, and North America. Clopidogrel monotherapy trials were conducted exclusively in Asia. The primary objective was to determine that ticagrelor or clopidogrel monotherapy is non-inferior to DAPT for the prevention of death, MI, or stroke. The major bleeding and NACE were the key secondary endpoints. The study definitively concluded that the efficacy of P2Y12 inhibitor monotherapy after a short course of DAPT is independent on the type of P2Y12 inhibitor used. Furthermore, the study clearly showed that ticagrelor monotherapy and clopidogrel monotherapy are more effective than DAPT in preventing death, MI, or stroke, and in reducing major bleeding and NACE in patients without an indication for oral anticoagulation undergoing PCI [5].

Gragnano et al. [6] found that P2Y12 monotherapy is more effective and equally safe compared to ASA in preventing coronary events in patients with coronary artery disease (Table 3). The data came from seven trials at the patient level [16,20,51,52,53,54,55]. This exhaustive individual patient data meta-analysis of randomized controlled trials included 24,325 participants with established CAD, of whom a considerable proportion received contemporary pharmaceutical invasive treatments. The results suggest a significant decrease in ischemic events with P2Y12 inhibitor monotherapy versus aspirin monotherapy for secondary cardiovascular prevention without increased bleeding risk. Patients who received a P2Y12 inhibitor had a 12% relative risk reduction in the primary composite endpoint of cardiovascular death, myocardial infarction, or stroke. This benefit was primarily attributable to a 23% relative reduction in heart attack and a secondary, but not significant, reduction in stroke.

This analysis challenges the established benefits of aspirin, particularly in the context of secondary prevention. It is well documented that aspirin is an effective antiplatelet agent; however, the potential for adverse effects must be considered. These include both intracranial and extracranial bleeding complications, which occur in a significant proportion of patients undergoing long-term aspirin therapy [56,57]. Furthermore, it remains uncertain whether these results would be replicated in contemporary trials implementing current secondary prevention strategies, since the pivotal trials of aspirin were conducted several decades ago. In this context, alternative approaches to the long-term management of patients with CAD are suggested by the availability of other antithrombotic agents with different antiplatelet effects [56,57,58]. Table 3 provides a comprehensive overview of the selected studies for the meta-analysis [6,16,20,51,52,53,54,55].

The results of meta-analyses may be inconsistent due to the way the data were analyzed [6,59]. The clinical relevance of P2Y12 monotherapy is questionable. This is the conclusion of six of the co-authors of the Gragnano meta-analysis [6], and they are well qualified to comment on this issue. 

For example, Charito et al. [48] conducted a meta-analysis of nine randomized trials involving 42,108 patients who were administered either aspirin (*n* = 21,065) or P2Y12 (*n* = 21,043) [20,52,53,59,60,61,62,63,64]. The study compared P2Y12 inhibitor monotherapy with aspirin for secondary prevention in patients with established cerebral, coronary, and peripheral arterial disease. In essence, the principal findings of this investigation may be encapsulated as follows: firstly, there was no discernible discrepancy in the incidence of all-cause mortality, cerebrovascular mortality, or stroke between the two groups of subjects (those treated with P2Y12 inhibitors and those treated with aspirin). Secondly, the relative risk of acute myocardial infarction appeared somewhat diminished in those receiving P2Y12 inhibitors compared to those receiving aspirin; the number needed to treat to prevent one myocardial infarction was estimated to be 244. Thirdly, the findings were observed to be consistent irrespective of the particular class of P2Y12 inhibitor administered. 

It is of significant importance to note that this is the inaugural comprehensive meta-analysis to include studies comparing ticagrelor monotherapy with aspirin monotherapy. Furthermore, the results from Chiarito et al. do not demonstrate any effect of P2Y12 type, aspirin dose, or duration of follow-up on the effect estimate. The authors posit that the progression of atherosclerosis and subsequent ischemic events share a common underlying pathophysiology across different arterial territories. In clinical practice, it is not uncommon to encounter patients who exhibit evidence of atherosclerosis in multiple vascular districts. Moreover, in patients undergoing secondary prevention, the occurrence of ischemic events in a different vascular territory from the initial event is commonly noted [65,66,67,68]. A reduction in the risk of myocardial infarcts with P2Y12 inhibition compared with aspirin was previously suggested only by a post hoc analysis of the CAPRIE study [53]. In a leave-one-out sensitivity analysis, the reduction in the risk of myocardial infarcts shown in the present analysis lost significance after the CAPRIE study was excluded. The neutral effect of P2Y12 inhibitor monotherapy on total and vascular mortality compared to aspirin monotherapy, with precise risk estimates, suggests that the beneficial treatment effect observed in MI does not translate into significant improvement in clinical outcomes. The authors found a similar risk of major bleeding in patients receiving a P2Y12 inhibitor and those receiving aspirin, whereas gastrointestinal bleeding was significantly lower in patients receiving a P2Y12 inhibitor. This could be explained in part by a direct injury to the gastric mucosa caused by aspirin, but not by P2Y12 inhibitors [53]. However, bleeding events stratified according to the source of bleeding were not consistently reported in all the included trials, which prevents us from drawing more robust conclusions. 

In patients with established atherosclerosis, P2Y12 inhibitor monotherapy is associated with a lower risk of myocardial infarction and a comparable risk of stroke compared with aspirin monotherapy. However, given the high number needed to treat to prevent one myocardial infarction and the lack of effect on all-cause and vascular death, the benefit in favor of P2Y12 inhibitor monotherapy is of questionable clinical relevance. In addition, there were no differences in ischemic or bleeding endpoints between patients who received only aspirin and those who received only a P2Y12 inhibitor [49,61].

**Figure 3:**
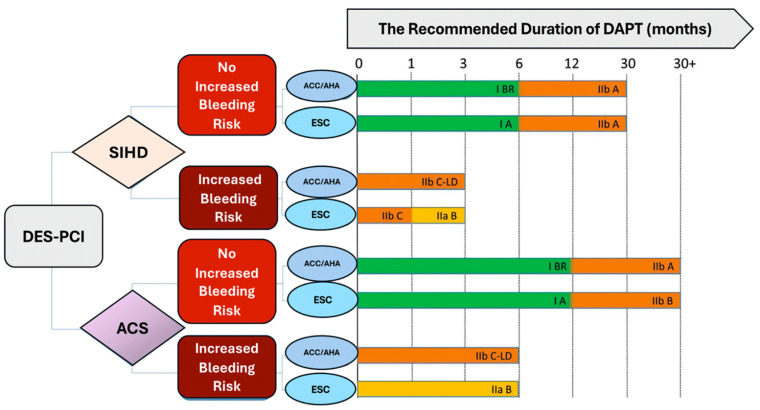
This guidance is intended to provide recommendations on the optimal duration of dual antiplatelet therapy following percutaneous coronary intervention with drug-eluting stents. The recommendations are classified as follows: Class I: Benefit much greater than risk; Class II: Benefit greater than risk, with conflicting evidence or opinion; Class IIa: Weight of evidence or opinion is in favor of usefulness; Class IIb: Usefulness is less well established. Level of evidence: A: Based on multiple randomized clinical trials. B or BR: Based on one or more randomized trials. C or C-LD: Based on nonrandomized observational studies. Abbreviations, ACC = American College of Cardiology; ACS = acute coronary syndrome; AHA = American Heart Association; DAPT = dual antiplatelet therapy; DES = drug-eluting stent; ESC = European Society of Cardiology; PCI = percutaneous coronary intervention; SIHD = stable ischemic heart disease [66,67,68,69].

In a recent meta-analysis, Ando and colleagues [69] confidently confirmed that, despite theoretical advantages, the clinical advantages of inhibiting the cyclooxygenase enzyme for secondary prevention outweigh those offered by inhibiting the P2Y12 receptor. The analysis included several studies and 73,126 patients [15,18,19,26,54,69,70,71,72,73,74,75,76,77,78,79,80] demonstrated that aspirin increases the risk of MI compared to P2Y12-I monotherapy (risk ratio: 1.32; 95% CI: 1.08–1.62) in line with the frequentist framework. This study definitively demonstrated that both monotherapies reduced major bleeding compared to DAPT. However, only P2Y12-I demonstrated equivalent efficacy in preventing MI. There were no significant differences observed in major bleeding, death, and other thrombotic outcomes. Based on point estimates, P2Y12-I monotherapy was associated with a lower risk for stent thrombosis and stroke. This finding was evident and unwavering in all models, including the fixed-effects model and the Bayesian framework, which demonstrated optimal convergence. P2Y12-I monotherapy was the clear winner, reducing all assessed outcomes more effectively than aspirin monotherapy [69] (Figure 3 and Table 4).

While meta-analyses represent a robust form of statistical methodology [5,6,48,49,50,69], there are differences between study-level meta-analyses [6,48,49,50,69] and patient-level meta-analyses [5] that address the results directly from data compiled from the individual randomized trials screened. Additionally, a significant number of authors arrive at disparate conclusions based on the studies conducted [6].

### 4.2. Thrombotic Complications Receptor Signaling: Emerging Evidence from RCTs and Meta-Analysis

It seems appropriate to investigate the potential of aspirin to inhibit platelet P2Y12 receptor signaling, given the central role of platelet P2Y12 signaling in thrombotic complications and the established association between aspirin and bleeding, particularly gastrointestinal bleeding [6,73,74,75,76,77,78,79,80,81,82]. Discontinuation of aspirin instead of the P2Y12 inhibitor may be a strategy to reduce bleeding while preserving ischemic protection [25,26,27]. A series of recently conducted clinical trials have examined the efficacy of P2Y12 inhibitor monotherapy, primarily utilizing ticagrelor and clopidogrel, in conjunction with a brief course of DAPT [15,16,17,18,19,20]. When evaluated individually, each study has limitations in design, power, or both that preclude definitive conclusions for practice [15,16,17,18,19,20]. A meta-analysis of aggregate data has demonstrated comparable ischemic and bleeding risks associated with P2Y12 inhibitor monotherapy when compared with continuing dual antiplatelet therapy (DAPT). However, the analysis did not examine the potential role of P2Y12 inhibitor type following the discontinuation of DAPT [49,83]. A previous patient-level meta-analysis found no evidence of treatment heterogeneity between clopidogrel and newer P2Y1/2 inhibitors [21]. However, only 2586 patients (22.2%) were on clopidogrel monotherapy, compared with 9048 patients (77.8%) on newer P2Y12 inhibitor monotherapy [21]. The updated meta-analysis includes nearly twice the number of patients treated with clopidogrel [21]. In the per-protocol analysis, clopidogrel monotherapy was associated with a significantly higher risk of the primary end point, with a 37% increase compared with aspirin and clopidogrel. In terms of numerical frequency, all three components of the primary endpoint were found to be more common with clopidogrel monotherapy than DAPT. While these results were consistent across all subgroups, the assessment of absolute risks suggests that the signal of injury may be more significant in patients with ACS. On the other hand, the bleeding benefit seen with clopidogrel alone and the absence of NACE suggest that this strategy involves a compromise between ischemic and bleeding events and may be justified in patients at risk of bleeding [84].

The recent meta-analysis by Valgimigli and colleagues [5] provides evidence that discontinuation of aspirin 1 to 3 months after PCI and switching to ticagrelor may be safe and at least as effective as standard DAPT. A relative margin of 15% on the HR scale was used to establish non-inferiority for the primary endpoint. The upper limits of the two-sided 95% CIs in the protocol and intention-to-treat analyses are consistent with a relative risk increase of no more than 6% compared with DAPT. It is important to consider the remaining potential for a small risk in the context of the significant reduction of major bleeding (53%) and non-acute cardiovascular events (26%), as observed in the data. Additionally, there was a nominally significant 28% lower risk of mortality with ticagrelor monotherapy. The significant reduction in major bleeding observed with this therapy may explain the mortality benefit [85,86].

**Figure 4:**
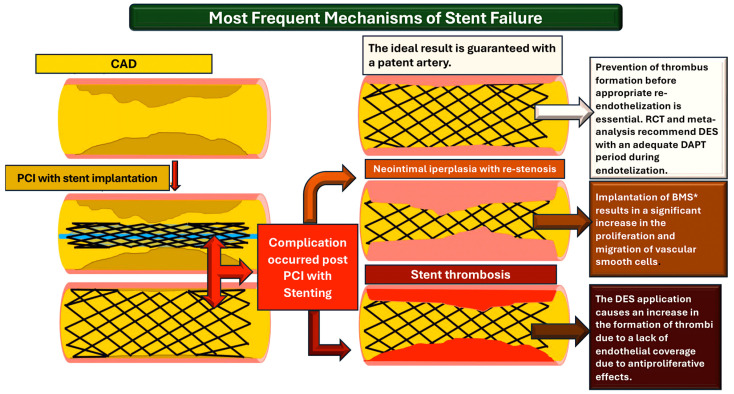
This figure illustrates the most frequent mechanisms of stent failure. The illustration outlines the PCI procedure with stent implantation for CAD, as well as the associated complications. Abbreviations, CAD; coronary artery disease; PCI, percutaneous coronary intervention [1,2,32,33,46,51,52,57,58,66,67,68,69,70,71]. * Not supported by current guidelines.

It is a common occurrence for patients undergoing PCI to exhibit high platelet reactivity when using clopidogrel [22]. This is associated with an increased risk of thrombotic complications [22,87]. Individuals with high platelet reactivity on clopidogrel may be at even greater risk if aspirin is withdrawn and there is no or minimal antiplatelet response. Ticagrelor provides deeper and more sustained P2Y12 inhibition than clopidogrel [22]. Due to the absence of pharmacodynamic and genetic data within the scope of this study, we are unable to determine whether high platelet reactivity while utilizing clopidogrel or CYP2C19 genotypes can account for the disparate outcomes associated with ticagrelor and clopidogrel monotherapy. The clopidogrel monotherapy trials were conducted in Japan or Korea. East Asian patients have a higher frequency of CYP2C19 loss-of-function alleles [33] and may be more prone to no or poor response to clopidogrel. In contrast, the East Asian population has a lower incidence of heart disease and a lower risk of atherothrombotic complications after PCI than the Caucasian population [88]. In a multicenter, randomized, controlled trial involving patients at high risk of bleeding, abbreviated dual antiplatelet therapy was found to be non-inferior to standard DAPT with respect to ischemic events, and superior with regard to bleeding [84,89]. The investigators had the discretion to select monotherapy type, and randomization was not stratified by antiplatelet monotherapy type. In the abbreviated therapy group, 53.9% of patients received clopidogrel monotherapy, which did not appear to result in an overall signal of harm compared with standard DAPT, in contrast with findings from the STOPDAPT-2 ACS trial by Watanabe et al. [26] Figure 4.

## 5. Future Direction

The study of antiplatelet drugs and platelet reactivity in response to specific applied therapies has led to the discovery of new avenues in research and clinical application (Table 5) [38,47,90,91,92,93,94].

Of particular interest are the novel antiplatelet agents under development, which target PAR1, PAR4, GPVI, Syk, and 12-LOX. Interestingly, the use of drugs that target PAR1 and PAR4 is a promising approach to regulating thrombin function. Thrombin is an extremely potent agonist that activates human platelets by proteolytic cleavage of PAR1 and PAR4, which are high- and low-affinity receptors, respectively. Two PAR1-specific antagonists have been licensed: vorapaxar and atopaxar [95,96]. The latest studies have examined the impact of triple antiplatelet therapy on cardiovascular death by combining vorapaxar with standard antiplatelet therapy (aspirin and clopidogrel). Patients undergoing triple therapy exhibited lower rates of cardiovascular death, but also experienced an uptick in intracranial hemorrhage [95,97,98]. Despite these findings, vorapaxar has been cleared for use in patients with a history of cardiovascular disease and no history of stroke. However, it must be used in conjunction with another platelet inhibitor. 

GPVI, a platelet-specific collagen receptor complexed with FcγR, plays a crucial role in regulating both platelet adhesion and activation [99,100]. GPVI inhibitors, including anti-GPVI antibodies and a soluble GPVI-Fc fusion protein called Revacept, have been developed as potential antiplatelet agents [99,100,101].

Spleen tyrosine kinase (Syk) is a key regulator of platelet activation downstream of tyrosine kinase-coupled receptors GPVI, C-type lectin-like receptor 2, and FcγRIIA. Despite prior investigations identifying Syk as a central regulator of platelet activation, deletion of Syk did not de facto cause increased bleeding [99,100]. A recent in vivo study by van Eeuwijk et al. [93] definitively demonstrated that platelet-specific Syk knockout mice exhibited a relatively mild hemostatic defect. However, these mice were strongly protected in a model of arterial thrombosis. Similar findings were observed when a selective Syk inhibitor, BI1002494, was administered to wild-type mice (Table 5) [94]. Syk inhibitors have also been investigated as a possible therapeutic option for heparin-induced thrombocytopenia. For example, PRT-060318 demonstrated efficacy in preventing the spontaneous formation of thrombi within the pulmonary vasculature and in maintaining platelet counts in a humanized FcγRIIA and platelet factor 4 mouse model (Table 5) [94].

LOXs (lipoxygenases) are a family of enzymes that may play a role in the oxygenation of polyunsaturated fatty acids. It is thought that they may generate a variety of active signaling molecules. 12-LOX, named for the ability of this family member to oxidize arachidonic acid at carbon 12, is found in both megakaryocytes and platelets, according to the latest evidence [95,102].

## 6. Conclusions

New research has identified a number of previously unknown mechanisms that either facilitate or impede signaling events downstream of receptor-mediated platelet activation. In several cases, it appears that disrupting these pathways can selectively inhibit thrombosis while leaving essential hemostatic processes largely intact. These pathways may be of significant interest as potential targets for the development of a new generation of antiplatelet agents. Other research has concentrated on developing innovative antiplatelet medications that target well-defined pathways such as P2Y12, PAR1, and GPVI.

It is a reasonable hypothesis that these agents could address certain shortcomings associated with existing therapies. Nevertheless, it remains uncertain whether these novel agents will be widely implemented in clinical practice. The current research is constrained by several limitations, including a lack of information regarding pharmacodynamic and genetic factors. This precludes us from reaching a definitive conclusion regarding the role of platelet activity in explaining the differences in treatment outcomes between ticagrelor and clopidogrel monotherapy.

Only in Japan and Korea have trials of clopidogrel monotherapy been undertaken. East Asian individuals exhibit a higher prevalence of CYP2C19 loss-of-function alleles, which predisposes them to a lack of or poor response to clopidogrel. In contrast, the prevalence of ischemic heart disease and the risk associated with post-PCI atherothrombotic complications are both significantly reduced among East Asian populations when compared with those of Caucasian populations [87]. A global clinical trial conducted among patients at high bleeding risk demonstrated the non-inferiority of the shortened DAPT regimen to that of the standard DAPT regimen for ischemic events and the superiority of the shortened DAPT regimen for bleeding [88,89].

It appears that a relatively high number of patients prescribed clopidogrel have an excess risk of thrombotic complications after PCI, which may be related to a tendency to high platelet reactivity while on clopidogrel [84,88,89]. In such cases, it may be helpful to explore the possibility of discontinuing aspirin in order to assess whether the effect of the antiplatelet treatment is maintained at a minimal level. It is also important to bear in mind that aspirin withdrawal may be at even greater risk in patients who are prone to high platelet reactivity during clopidogrel use. Ticagrelor is believed to be a more potent P2Y12 receptor inhibitor than clopidogrel [89].

Finally, in patients with CAD, P2Y12 inhibitor monotherapy was associated with a lower risk of cardiovascular death, MI, and stroke compared with aspirin monotherapy [5,6,49,50,56,69]. This association is mainly attributed to a lower risk of myocardial infarction, which resulted in a decreased risk of non-acute coronary syndrome events. The incidences of major bleeding and gastrointestinal bleeding were similar in both groups. Nevertheless, the incidence of hemorrhagic stroke was found to be lower in patients receiving monotherapy with a P2Y12 inhibitor [84,85,89]. The available randomized data indicates that long-term P2Y12 inhibitor therapy may be a more appropriate choice than aspirin monotherapy for secondary prevention in patients with CAD [5,6,49,50,56,69]. Central Illustration:

**Figure:**
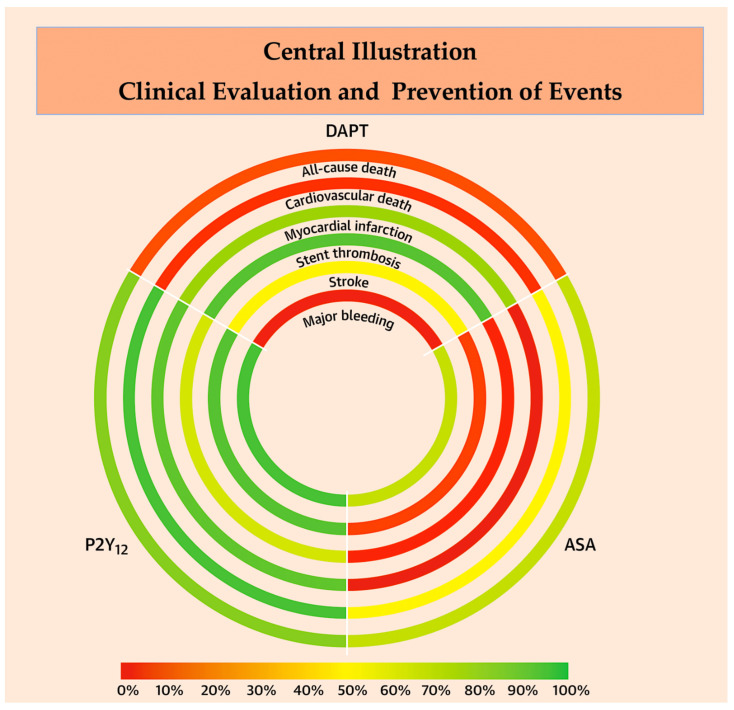
**Central Illustration**: The illustration demonstrated that the antiplatelets treatment therapies under discussion had been ranked as the best for preventing events based on data from 73,126 patients included in 19 RCTs analyzed by Ando G. et al. [69] The rank-heat plot, seen below, illustrates treatment ranking across outcomes for aspirin vs. P2Y12 inhibitor monotherapy. Each circle represents one outcome, and each sector represents each intervention. In this way, the three sectors are colored in accordance with the treatment ranking at the corresponding outcome. Green and red correspond to the highest (100%) and the lowest (0%) ranking statistic values, respectively. Values near the scale middle are yellow. Abbreviations; ASA, aspirin; DAPT, dual antiplatelet therapy; RR: risk ratio.

## Figures and Tables

**Table 1:** P2Y12 receptor inhibitor therapies.

Drug	Method of Delivery	Evolutionary Step	Benefits	Drawbacks	Comment
P2Y12 inhibitors. Thienopyridines. Clopidogrel bisulfate	Oral	FDA (17 November 1997) and EMEA (1998) approved	Clopidogrel is a widely used drug by cardiologists, emergency department physicians, family practice clinicians, and internists.	- Genetic polymorphism for one or both alleles of the CYP2C19 enzyme. - Risk factors for bleeding include age older than 75 years - Use of medications (e.g., non-steroid anti-inflammatory agents or warfarin) that can increase the risk of bleeding.	Patients with any loss of function allele will not effectively metabolize clopidogrel, leading to the inability to inhibit platelet activity.
P2Y12 inhibitors. Thienopyridines. Prasugrel	Oral	EMEA and FDA approved	- More potent antiplatelet action with earlier onset and offset compared with clopidogrel - CYP450 genetic polymorphism has no impact on the antiplatelet effect. - PPIs do not significantly interact with coumarin derivatives.	This medication may carry a higher risk of bleeding when compared to clopidogrel.	Use cautiously in patients with stroke, TIA, aged 75 years, weighing <60 kg.
P2Y12 inhibitors. Thienopyridines. Elinogrel	Oral; iv	Discontinued in January 2012 by Novartis	- Antiplatelet action unaffected by common genetic polymorphisms of CYP450 - No incremental bleeding risk when added to dual antiplatelet therapy (aspirin and clopidogrel)	Elinogrel therapy was associated with an increased incidence of dyspnea and incidence of elevated liver transaminases.	The potential use of this as an adjunct or alternative to standard antiplatelet therapy, such as aspirin and/or clopidogrel, was not pursued.
P2Y12 inhibitors. Cyclopentyl triazolo- Pyrimidine. Ticagrelor	Oral	EMEA and FDA approved	Exhibits a faster onset and offset of antiplatelet action compared to clopidogrel.	Possible side effects include dyspnea, bradycardia, and elevated uric acid levels.	- Use cautiously in patients with stroke - Can be used as an alternative antiplatelet for patients who do not respond to clopidogrel.
P2Y12 inhibitors. ATP analogs. Cangrelor	Iv	EMEA and FDA approved	Exhibits a faster onset and offset of antiplatelet action compared to clopidogrel, prasugrel, and ticagrelor.	- Administer intravenously. - Side effects: dyspnea - Minor bleeding occurs more frequently with this medication than with clopidogrel.	This is a promising adjunct treatment to fibrinolysis.

**Table 2:** Patients with ticagrelor or clopidogrel monotherapy or DAPT in the Valgimigli patient-level meta-analysis.

RCT Author/Reference	Ticagrelor Monotherapy (*n* = 8790)	Aspirin + P2Y12 Inhibitor (*n* = 8791)	Clopidogrel Monotherapy (*n* = 4110)	Aspirin + P2Y12 Inhibitor (*n* = 4144)	Comment Ticagrelor (HR, 0.89; 95% CI, 0.74–1.06; *p* for Non-Inferiority = 0.004) Clopidogrel (HR, 0.89; 95% CI, 0.74–1.06; *p* for Non-Inferiority = 0.004)
Franzone et al. [33]	3753 (42.7)	3756 (42.7)	0	0	Ticagrelor monotherapy after 1 month of DAPT non-inferior to conventional treatment in the prevention of ischemic events. Superior efficacy not demonstrated. Reduction in the risk of major bleeding in comparison to conventional treatment not observed. *p* = 0.98
Hahn et al. [34]	273 (3.1)	263 (3.0)	1122 (27.3)	1143 (27.6)	P2Y12 inhibitor monotherapy after 3 months of DAPT resulted in non-inferior rates of MACCEs compared with prolonged DAPT among patients undergoing PCI. (*p* = 0.007)
Watanabe et al. [35]	0	0	1496 (36.4)	1507 (36.4)	1 month clopidogrel monotherapy non-inferiority (*p* < 0.001) and superiority (*p* = 0.04) to DAPT. Clopidogrel monotherapy more effective than 12 months of DAPT in reducing the rate of a composite of cardiovascular and bleeding events. (HR, 0.26 [95% CI, 0.11–0.64]; *p* = 0.004 for superiority)
Watanabe et al. [36]	0	0	1492 (36.3)	1494 (36.0)	No net clinical benefit with clopidogrel monotherapy after 1 to 2 months of DAPT. Increase in cardiovascular events and decrease in bleeding events (HR, 1.50 [95% CI, 0.99–2.26]) vs. (HR, 0.46 [95% CI, 0.23–0.94]).
Kim et al. [37]	1499 (17.1)	1505 (17.1)	0	0	Ticagrelor monotherapy after 3 months of DAPT, modest but statistically significant reduction in a composite outcome of major bleeding and cardiovascular events compared with ticagrelor-based 12-month dual antiplatelet therapy (HR, 0.69 [95% CI, 0.45 to 1.06]; *p* = 0.09).
Mehran et al. [38]	3265 (37.1)	3267 (37.2)	0	0	After 3 months of DAPT, ticagrelor monotherapy was associated with a lower incidence of clinically relevant bleeding than ticagrelor plus aspirin (*p* < 0.001), with no higher risk of death, myocardial infarction, or stroke (*p* < 0.001 for non-inferiority)

Abbreviations: ACS, acute coronary syndrome; CI, confidence interval; DAPT, dual antiplatelet therapy; DES, drug-eluting stent; HR, hazard ratio; MACCE, major cardiac cerebrovascular event; PCI, percutaneous coronary intervention; Pts, patients [1,33,34,35,36,37,38].

**Table 3:** RCT evaluation of DAPT vs. SAPT in the Gragnano study-level meta-analysis.

RCT Acronym/Reference	Item	Highlights Findings	Comment
ASCET [39]	Platelet function study clopidogrel vs. ASA (*n* = 1001)	73 death/MI/stroke events. No difference.	Not suitable for clinical events.
CADET [40]	Thrombogenicity study clopidogrel vs. ASA (*n* = 184)	No variables showed any differences.	Not suitable for clinical events
CAPRIE [41]	Clinical trial clopidogrel vs. ASA (*n* = 19,185) Recent MI subgroup (*n* = 6302) Stroke subgroup (*n* = 6431) PAD subgroup (*n* = 6452)	No differences were found in the recent (</= 35 day) MI subgroup or stroke subgroup. An additional post hoc subgroup was created, consisting of 2144 patients from the stroke and PAD subgroups who had a distant past history of MI, resulting in a larger 8446 patient subgroup with recent or distant MI.	The PANTHER meta-analysis utilized a post hoc subgroup of patients enrolled 28 years ago from subgroups of a trial that demonstrated no significant difference for patients with recent myocardial infarction or stroke.
DACAB [42]	SVG patency trial DAPT vs. ticagrelor vs. ASA (*n* = 500)	There were no significant differences in the occurrence of death, myocardial infarction, or stroke events between the ticagrelor and ASA subgroups, with a total of 13 events.	Not suitable for clinical events.
GLOBAL LEADERS [43]	Clinical trial 1 mo ^†^ DAPT/23 mo ticagrelor vs. 12 mo ^†^ DAPT/12 mo ^††^ ASA (*n* = 15,968)	There was no difference in the 2-year endpoint or in the landmark analysis when comparing ticagrelor to ASA after 1 year. However, the GLASSY subgroup analysis of the 20 top enrolling sites (*n* = 7585) demonstrated a lower MI rate after 1 year with no significant difference in efficacy between the two treatments.	Post hoc subgroup (*n* = 7065) of a post hoc subgroup (GLASSY) of a negative trial was used for the PANTHER meta-analysis.
HOST-EXAM [44]	Clinical trial clopidogrel vs. ASA after 6–18 mo ^†^ DAPT for PCI N = 5438	The study unequivocally demonstrates that the use of clopidogrel leads to a significantly lower incidence of adverse events, including all-cause death, myocardial infarction, stroke, readmission for acute coronary syndrome, and bleeding of type 3 or greater according to the Bleeding Academic Research Consortium (5.7% vs. 7.7%; HR: 0.73; 95% CI: 0.59–0.90; *p* = 0.0035).	No differences in death, MI, or stent thrombosis rates were observed. The reduced rates of stroke, readmission for ACS, and bleeding were attributed to the benefit.
TICAB [45]	Post-CABG clinical trial ticagrelor vs. ASA (*n* = 1859)	No differences were observed in the 117 death/MI/stroke events.	The study was stopped prematurely due to futility. Additionally, the ticagrelor group had a higher event rate.

Abbreviations; ACS, acute coronary syndrome; ASA, aspirin; BARC, Bleeding Academic Research Consortium; ASCET, Aspirin Non-responsiveness and Clopidogrel Clinical Endpoint Trial; CABG, coronary artery bypass grafting; CADET, Clopidogrel and Aspirin: Determination of the Effects on Thrombogenicity; CAPRIE, Clopidogrel Versus Aspirin in Patients at Risk of Ischaemic Events; CI, confidence interval; DACAB, Different Antiplatelet Therapy Strategy After Coronary Artery Bypass Graft Surgery; DAPT, dual antiplatelet therapy; GLASSY, GLOBAL LEADERS Adjudication Sub-Study; GLOBAL LEADERS, Comparative Effectiveness of 1 Month of Ticagrelor Plus Aspirin Followed by Ticagrelor Monotherapy Versus a Current-Day Intensive Dual Antiplatelet Therapy in All-Comers Patients Undergoing Percutaneous Coronary Intervention With Bivalirudin and BioMatrix Family Drug-Eluting Stent Use; HOST-EXAM, Harmonizing Optimal Strategy for Treatment of Coronary Artery Stenosis-Extended Antiplatelet Monotherapy (mo) ^†^; HR, hazard ratio, MI, myocardial infarction; PAD, peripheral artery disease; PANTHER, P2Y12 inhibitor or aspirin monotherapy; PCI, percutaneous coronary intervention; RCT, randomized clinical trial; SVG, saphenous vein graft; TICAB, Ticagrelor in Coronary Artery Bypass. GLOBAL LEADERS Adjudication Sub-Study), alive patients at 1 year after randomization who did not experience myocardial infarction, stroke, or Bleeding Academic Research Consortium type 3 or type 5 bleeding and who were not lost to follow-up during the first year after enrollment were included ^††^ [2,39,40,41,42,43,44,45].

**Table 4:** Meta-analysis evaluating DAPT vs. SAPT.

First Author, Year (Ref. #)	N	Endpoints/Findings	*p* Value/I2	HR/OR
Valgimigli et al. 2024 [5]	DAPT (12,403) P2Y12 inhibitor plus aspirin vs. SAPT (11,991) SAPT Ticagrelor 8292 Clopidogrel 3654 Prasugrel 45	Non-inferiority of ticagrelor or clopidogrel monotherapy vs. DAPT on the composite of death, MI, or stroke Ticagrelor monotherapy non-inferior to DAPT for all-cause death, MI, or stroke and superior for major bleeding and NACE	*p* = 0.004 *p* < 0.001	HR: 0.89; HR: 1.00
Gragnano et al. 2023 [6]	SAPT Ticagrelor 4633 Clopidogrel 7545 Aspirin 12,147	Composite of cardiovascular death, MI, and stroke Secondary endpoint major bleeding and NACE P2Y12 inhibitor monotherapy might be preferred over aspirin monotherapy for long-term secondary prevention	*p* = 0.012 *p* < 0.001 for IM *p* = 0.23 for major bleeding	HR: 0.88 HR: 0.77 for IM
Ando et al. 2022 [69]	73,126 DAPT discontinued with aspirin or P2Y12-I vs. DAPT prolonged with aspirin vs. P2Y12-I	All-cause death; MI; major bleeding	-	HR: I.19 vs. 1.33 vs. 1.10 aspirin vs. P2Y12-I monotherapies
Lau et al. 2022 [49]	20,915 short DAPT ^†^ 20,949 prolonged DAPT	Stent thrombosis (ST) and bleeding. DAPT ≤ 3 months followed by SAPT reduces bleeding and is not associated with an increase in ST.	*p* = 0.26 *p* < 0.0001	HR: 1.17 HR: 0.65
Giacoppo et al. 2021 [50]	DAPT (16,088); Short DAPT (16,057) vs. P2Y12I or Aspirin	ST and bleeding. 1–3 months of DAPT followed by P2Y12I SAPT lower major bleeding and similar stent thrombosis, all-cause death, myocardial infarction, and stroke compared with prolonged DAPT.	*p* = 0.289 *p* = 0.850	HR: 1.19 HR: 0.63
Charito et al. 2020 [46]	P2Y12I inhibitor (21,043) Aspirin (21,065)	MI and stroke P2Y12I monotherapy is associated with a risk reduction for MI and a comparable risk of stroke	I2 = 10.9% I2 = 34.5%	OR: 0.81 OR: 0.93

Abbreviations; DAPT, dual antiplatelet therapy; HR, hazard ratio; MI, myocardial infarction; N, number of patients; NACE, net adverse clinical events; P2Y12I, P2Y12 inhibitor; SAPT, single antiplatelet therapy; ST; stent thrombosis. ^†^ Short DAPT discontinued vs. monotherapy.

**Table 5:** Selection of novel antiplatelet agents currently under development.

Target	Agent	Reference
P2Y1/P2Y12	GLS-409	[38]
PAR1	PZ-128	[90]
PAR4	BMS-986120	[91]
GPVI	Troα6, Troα10	[92]
Syk	BI1002494, PRT-060318	[93,94]
12-LOX	ML-355	[95]

Abbreviations: 12-LOX indicates 12-lipoxygenase; GP, glycoprotein; PAR, protease-activated receptor; Syk, spleen tyrosine kinase; Tro, trowaglerix [78,87,88,89,90,91,92].

## Data Availability

Not applicable.
